# Epithelial sodium channels (ENaC) produce extracellular positive DC potentials in the retinal neuroepithelium

**DOI:** 10.1016/j.dib.2015.11.068

**Published:** 2015-12-14

**Authors:** Masayuki Yamashita

**Affiliations:** Center for Medical Science, International University of Health and Welfare, 2600-1 Kitakanemaru, Ohtawara 324-8501, Japan

## Abstract

Positive DC (direct current) potentials were measured in the extracellular space in the retinal neuroepithelium of chick embryos. The positive DC potential was suppressed by amiloride, a blocker for epithelial Na^+^ channels (ENaC). Amiloride also decreased the resistance of the extracellular space as measured by passing a constant current through a microelectrode. The positive DC potential is necessary for the guidance of retinal ganglion cell axons [1].

**Specifications Table**

**Table:** 

Subject area	*Neuroscience*
More specific subject area	*Development, Physiology*
Type of data	*Figure*
How data was acquired	*Electrophysiological recordings with microelectrodes*
Data format	*Raw*
Experimental factors	*Isolated optic cups from chick embryos*
Experimental features	*Recording extracellular potentials from retinal neuroepithelium*
Data source location	*Department of Physiology 1, Nara Medical University, Shijo-cho 840, Kashihara, Japan*
Data accessibility	*Data is with this article*

**Value of the data**

• Presents novel roles for epithelial Na^+^ channels.
• Offers physiological characteristics of the neuroepithelium.
• Of interest for developmental neurobiologists.

## 1 Data

Neuroepithelial cells have a polarized structure: their apical process faces the ventricle, while the furthest portion of their basal process makes contact with the basement membrane. From the apical side, Na^+^ ions enter the neuroepithelial cells through amiloride-sensitive epithelial Na^+^ channels (ENaC), and are extruded by Na^+^–K^+^ pumps in the basal region to establish a positive DC potential inside the neuroepithelium [2]. Retinal neuroepithelial cells have a similarly polarized structure, in which the apical (outer) process faces the lumen that is continuous with the ventricle, and the basal (inner) process makes contact with the inner limiting membrane.

**Fig. 1:**
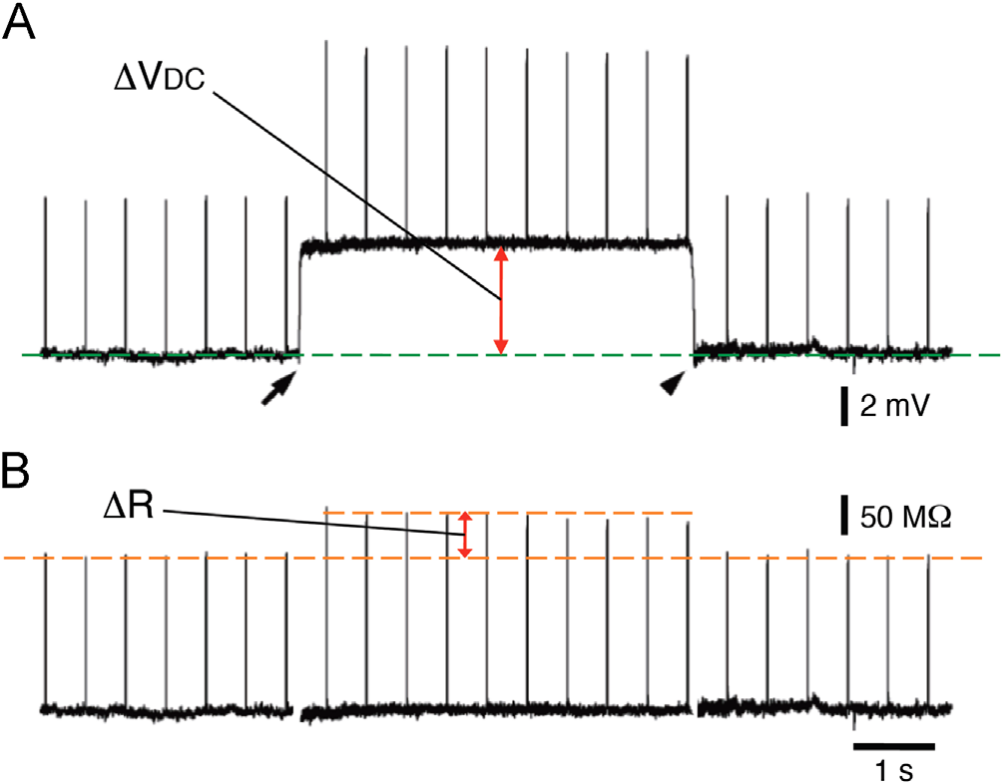
(A) A positive DC potential (ΔVDC). The inner limiting membrane of a retinal neuroepithelium was penetrated with a microelectrode (arrow). The extracellular potential was recorded immediately inside the inner limiting membrane. Then it was withdrawn from the retina (arrowhead). (B) An increase in the resistance between the electrode and the bath solution (ΔR). Current pulses (40 pA, 5 ms in duration) were passed through the electrode at 0.5 s-interval to monitor the resistance. Δ*R* is obtained by subtracting ΔVDC.

**Fig. 2:**
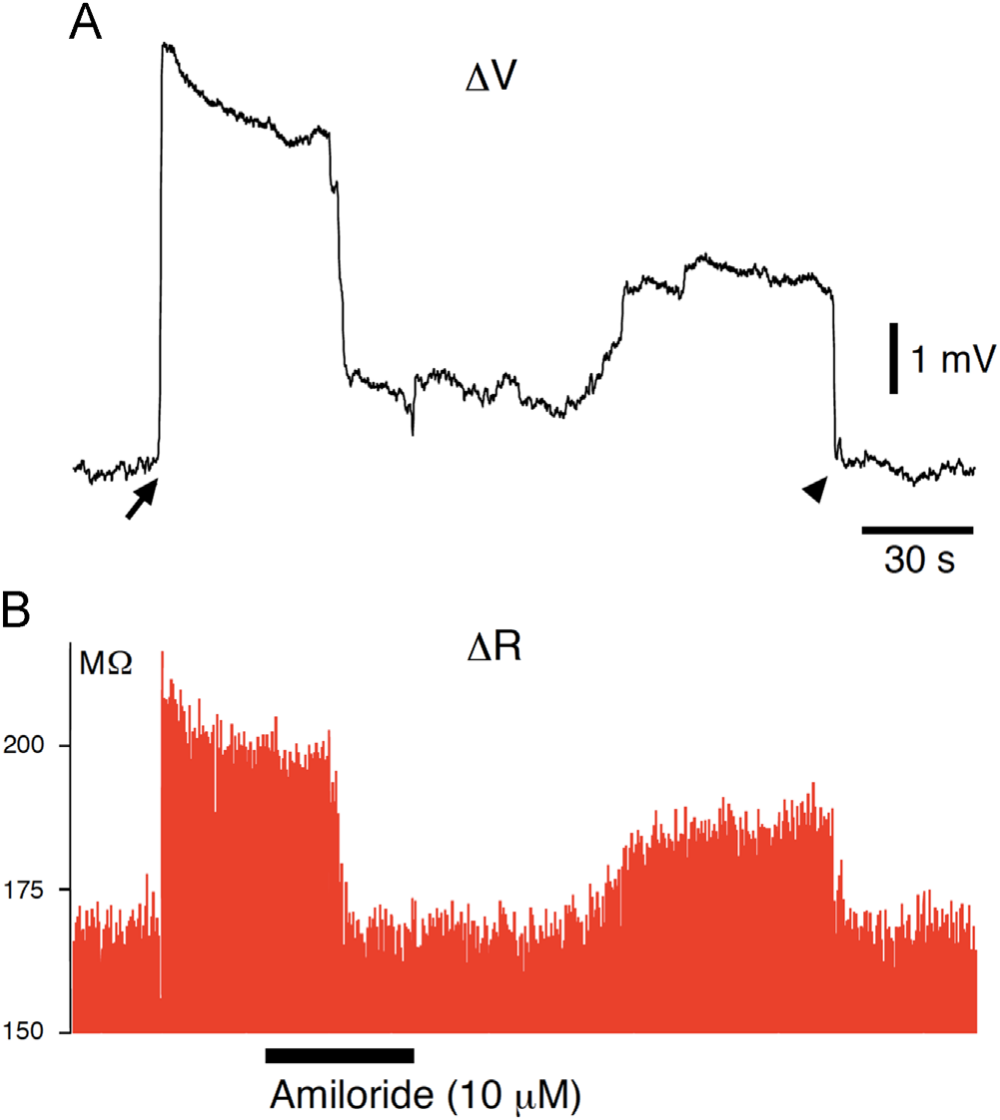
(A) Extracellular positive DC potential (Δ*V*) was reversibly suppressed by amiloride (10 μM). The inner limiting membrane was penetrated with a microelectrode (arrow), and withdrawn (arrowhead). Amiloride was bath-applied during the period indicated by the bar in the bottom. (B) The resistance between the electrode and the bath solution (Δ*R*) was also reduced reversibly.

Upon penetration of the inner limiting membrane of a retinal neuroepithelium from the vitreous side with a microelectrode, a positive DC potential was recorded (Fig. 1A, ΔVDC), with an increase in the resistance between the electrode and the bath solution (Fig. 1B, Δ*R*). The positive DC potential was suppressed by amiloride (10 μM, Fig. 2A). Amiloride also decreased the extracellular resistance to the level before the penetration (Fig. 2B). The amplitude of the positive DC potential was larger and the extracellular resistance was higher in the peripheral regions of the retina than in the central region, making a voltage gradient [1]. The axons of newborn retinal ganglion cells grow along this voltage gradient *in vivo* [1] and *in vitro* [3]. The data presented in this article are the supplementary materials of [1].

## 2 Experimental design, materials and methods

### 2.1 Preparation of retina

The optic cup was isolated from a chick embryo incubated for three days (E3) at 38 °C. The optic cup was positioned on the bottom of a recording chamber (volume, 0.2 mL) with the inner side up. The recording chamber was mounted on the fixed stage of an upright microscope (BX51WI, Olympus, Tokyo, Japan) under a water immersion objective (100×), and was perfused at 2 mL/min with a normal bath solution containing (mM); 137 NaCl, 5 KCl, 2.5 CaCl_2_, 1 MgCl_2_, 10 HEPES, 22 glucose, buffered to pH 7.3 by adding NaOH.

### 2.2 Electrical recording

Extracellular potentials were recorded immediately inside the inner limiting membrane of the retinal neuroepithelium with a glass microelectrode filled with 2 M NaCl (electrode resistance: 108–200 MΩ) using a conventional preamplifier for intracellular recording. Current pulses (40 pA, 5 ms in duration) were passed through the electrode at 0.5 s-interval to monitor the resistance between the electrode and the bath solution. Recordings were made at 36–38 °C. Details of electrical recording are described in [4].
